# Establishment of lung adenocarcinoma classification and risk model based on necroptosis-related genes

**DOI:** 10.3389/fgene.2022.1037011

**Published:** 2022-11-14

**Authors:** Guodong Wu, Dingwei Feng, Ziyu Zhang, Gao Zhang, Wei Zhang

**Affiliations:** ^1^ Department of Thoracic and Cardiovascular Surgery, The First Hospital of Fangshan District, Beijing, China; ^2^ Department of Thoracic Surgery, Beijing Yanhua Hospital, Beijing, China

**Keywords:** LUAD, necroptosis, immune microenvironment, risk model, immunotherapy

## Abstract

Lung adenocarcinoma (LUAD) is the most widely known histological subtype of lung cancer. Its classification is significant for the characteristic evaluation of patients. The aim of this research is to assess the categorization of LUAD and its risk model based on necroptosis and to investigate its potential regulatory mechanisms for diagnosing and treating LUAD. According to the expression profile data along with the clinical information related to LUAD from The Cancer Genome Atlas (TCGA) and Gene Expression Omnibus (GEO), we constructed a consistency matrix through consistency clustering, and used the ConsensusClusterPlus as the measurement distance to cluster and subtype the samples, and performed gene set enrichment analysis and immune infiltration analysis. Least absolute shrinkage and selection operator (Lasso) regression was utilized for obtaining prognostic significant necroptosis phenotype-related genes. Finally, we measured each patient’s riskscore (RS) and build a risk model, and predicted the effect of immunotherapy for different groups of risk factors in the model. Three molecular subtypes of LUAD were obtained by cluster analysis of necroptosis-related genes in LUAD samples. Compared with C1, C3 had a better prognosis and higher immune cell infiltration. The prognosis of the C1 subtype was poor and had a high clinical grade. The proportion of Stage II, Stage III, and Stage IV was much more in comparison with that of the other two subtypes. TP53 gene had a high mutation frequency in the C1 subtype. Gene Set Enrichment Analysis (GSEA) indicated that the aberrant pathways in the C1 and C3 subtypes mainly included some cell cycle-related pathways. In addition, seven genes were identified as related genes of necroptosis phenotype affecting prognosis. High RS had a poor prognosis, while low RS had a good prognosis. The RS was verified to have a strong ability to predict survival. LUAD can be classified by the genes linked with cell necrosis and apoptosis. The difference among various types is helpful to deepen the understanding of LUAD. In addition, a risk model was constructed based. In conclusion, this study provides potential detection targets and treatment methods for LUAD from a new perspective.

## Introduction

Lung cancer has the highest death rate around the globe (Bray et al., 2018). Its most widely known histological subtype is the Lung adenocarcinoma (LUAD), making up about 50% of the total lung cancer cases. It has a high risk of distant metastasis at each stage (Shi et al., 2016) and is linked with increased malignancy and a worse prognosis (Gong et al., 2019; Zhou et al., 2019). LUAD treatment is based on grade and stage and is mainly determined by the evaluation of tumor histology and patient characteristics by pathologists (Wei et al., 2019). The prognosis of lung cancer is unsatisfactory even though there has been improvement in its present treatment approach (chemotherapy, surgical resection, radiotherapy, immunotherapy, and molecular targeted therapy). Even at present, the 5-year survival rate of lung cancer patients is only 4%–17%, while the 5-year survival rate of metastatic tumor patients is <5% (Hirsch et al., 2017; Arbour and Riely, 2019; Anusewicz et al., 2020). Consequently, it is very important to diagnose this disease on time along with a detailed and precise risk assessment. Most of the risk assessment and monitoring tools that are being used at present for lung cancer use the clinical features and pathological parameters, among these the most widely used approach, is TNM stratification. Though, the current tumor-node-metastasis (TNM) models are usually linked with limited confidence in lung cancer prognosis prediction, which is composed of great heterogeneity among individuals. Therefore, it is necessary to coordinate the clinicopathological features of the genome when evaluating the survival prognosis of individuals.

Necroptosis, a kind of programmed necrotic cell apoptosis, is the gatekeeper of the host against pathogen invasion. It is a recently found type of programmed cell death that unlike apoptosis is unrelated to caspase (Robinson et al., 2019). The morphological manifestations of necroptosis are cell rounding and swelling, explosive rupture of the cell membrane, cell membrane perforation, mitochondrial dysfunction, and loss of mitochondrial membrane potential (Nikoletopoulou et al., 2013). During the inhibition or low level of caspase-8, receptor-interacting protein 1 (RIP1) can use receptor-interacting protein 3 (RIP3) to develop the complex of RIP1-RIP3, therefore, stimulating the mixed spectrum of pseudokinases. Phosphorylation of mixed-lineage kinase domain-like protein (MLKL) occurs to synthesize necrotic bodies, leading to necroptosis (Vandenabeele et al., 2010). The necroptosis imbalance is also a key factor in many inflammatory diseases. Necroptosis is known to have both positive and negative effects, and it has a complicated link with cancer. Even though research shows that upon the blockage of apoptosis, necroptosis can inhibit tumor growth as well as metastasis, however, its key regulators will promote tumor growth and metastasis (Liu et al., 2021). Increasing evidence shows that necroptosis has the ability to inhibit the growth and metastasis of tumors, so it can be used as a potential method to treat cancer (Li et al., 2020a; Park et al., 2020; Tan et al., 2020). These reports have highlighted the significant involvement of necroptosis in tumorigenesis and metastasis, suggesting the potential of targeting necroptosis as a new tumor classification and treatment.

In this study, we identified stable molecular subtypes by consensus clustering using genes associated with cell necroptosis and compared the clinicopathological features, mutation features, immune features, and pathway features among subtypes. Finally, the genes linked with the prognosis score and necroptosis were found by expression difference analysis and Lasso. Then, the risk model and clinical prognosis model were constructed, which could assist in the personalized treatment of LUAD patients.

## Materials and methods

### Collection and processing of data

The mutation, as well as RNA-Seq data of LUAD, were taken from The Cancer Genome Atlas (TCGA, http://cancergenome.nih.gov/abouttcga) using TCGA GDC API. In the RNA-Seq data, we removed the samples with no information regarding clinical follow-up, survival time, and status. After selecting, a total of 500 samples of primary LUAD were obtained. Then, the Ensembl in the data was changed into a Gene symbol, and the expression of numerous gene symbols was considered the mid-value. The expression data of the GSE72094 and GSE31210 datasets were taken from the Gene Expression Omnibus (GEO) (https://www.ncbi.nlm.nih.gov/geo/). 398 and 226 LUAD samples were included respectively after selection. For the above GEO data set, the annotation information of the corresponding chip platform was downloaded. According to the annotation information, the probe was mapped to the gene, and the single probe matching numerous genes was eliminated. When a gene was matched with multiple probes, we considered the median as the gene expression value. The current study utilized the TCGA as the training set, and GSE72094 and GSE31210 data sets were utilized as independent verification sets. In addition, our necroptosis-related genes came from previous study (Xin et al., 2022), with a total of 74 genes.

### Molecular typing of necroptosis-related genes

Univariate Cox analysis by Cox function in the R package highlighted the genes substantially linked with LUAD prognosis (*p* < 0.05). A consistency matrix was constructed by ConsensusClusterPlus (Wilkerson and Hayes, 2010) to cluster and divide the samples according to these genes. The molecular subtypes of samples were provided by the expression data of genes linked with necroptosis. We carried out 500 bootstraps with the “PAM” algorithm and “1-Pearson correlation” as the distance measurement. Each bootstrap had 80% of the subjects in the training set. The cluster number was set from 2 to 10. The best division was done by measuring the consistency matrix and the cumulative distribution function (CDF), and we got the molecular subtypes of the samples.

### Establishing of risk model

The differentially expressed necroptosis genes (false discovery rate (FDR) < 0.05 and |log2fold change (FC)| >1) were selected by limma package in molecular subtypes. Afterward, we chose genes that were expressed differentially and had a significant prognosis (*p* < 0.05). The proportion of genes was further reduced by Least absolute shrinkage and selection operator (Lasso) regression, and major prognostic genes related to the phenotype of necroptosis were obtained. By creating a penalty function, it can obtain a more precise model by compressing some coefficients and setting others to zero. To process data with complicated collinearity is a biased estimation that yet preserves the benefit of subset contraction. It makes variable selection during parameter estimation possible and improves the way multicollinearity in regression analysis is dealt with. The risk model was subsequently created. The prognosis risk score (RS) for individual patients was determined with: RS = Σ βi × Expi.

Expi is referred to as the level of expression of genes linked with the prognosis of necroptosis phenotype, β is referred to as the Cox regression coefficient of the corresponding gene. The patients were sorted into RS-high and RS-low groups according to the threshold “classification.” We drew the survival curve by the Kaplan-Meier method for prognosis analysis, and the significance of the difference was found with the help of the log-rank test.

### Prediction of immunotherapy effect

The Tumor Immune Dysfunction and Exclusion (TIDE) algorithm (Jiang et al., 2018) was employed for verification of the impact that immune microenvironment score (IMS) has on the prediction of clinical response of immune checkpoint inhibitors (ICIs). TIDE algorithm is a calculation method for predicting immune checkpoint blockade (ICB) reactivity by using a gene expression profile. It evaluates three types of cells that inhibit the infiltration of T cells in tumors, including myeloid-derived suppressor cells (MDSCs), tumor-associated fibroblasts (TAF), and the M2 subtypes of tumor-associated macrophages (TAMs), as well as two distinct subtypes of tumor immune escape mechanisms, including tumor-infiltrating cytotoxic T lymphocytes (CTL) dysfunction score and CTL immunosuppressive factor rejection score. The higher TIDE prediction score indicated an increased likelihood of immune escape, showing immunotherapy to be less beneficial for patients.

### Gene set enrichment analysis

For understanding the pathways of various biological mechanisms in a variety of molecular subtypes, GSEA was employed for pathway analysis. We utilized all candidate gene sets present in the Hallmark (Liberzon et al., 2015) for GSEA. FDR <0.05 was taken as a significant enrichment.

### Immune infiltration analysis

Cell type Identification By Estimating Relative Subsets Of RNA Transcripts (CIBERSORT) algorithm (Chen et al., 2018) (https://cibersort.stanford.edu/) was used for the quantification of 22 immune cells’ relative abundance in LUAD. Simultaneously, the number of immune cells was measured with the help of the Estimation of Stromal and Immune cells in Malignant Tumor tissues utilizing Expression data (ESTIMATE) software (Yoshihara et al., 2013).

### Statistical analysis

All R packages and statistical analysis were conducted in R software (4.1.1). Parameters with no specific indication were default. Statistical methods were indicated in the figure legends. *p* < 0.05 was considered as significant. ns, no significance. *p < 0.05, **p < 0.01, ***p < 0.001, ****p < 0.0001.

## Results

### Molecular typing on the basis of genes related to necroptosis

**FIGURE 1:**
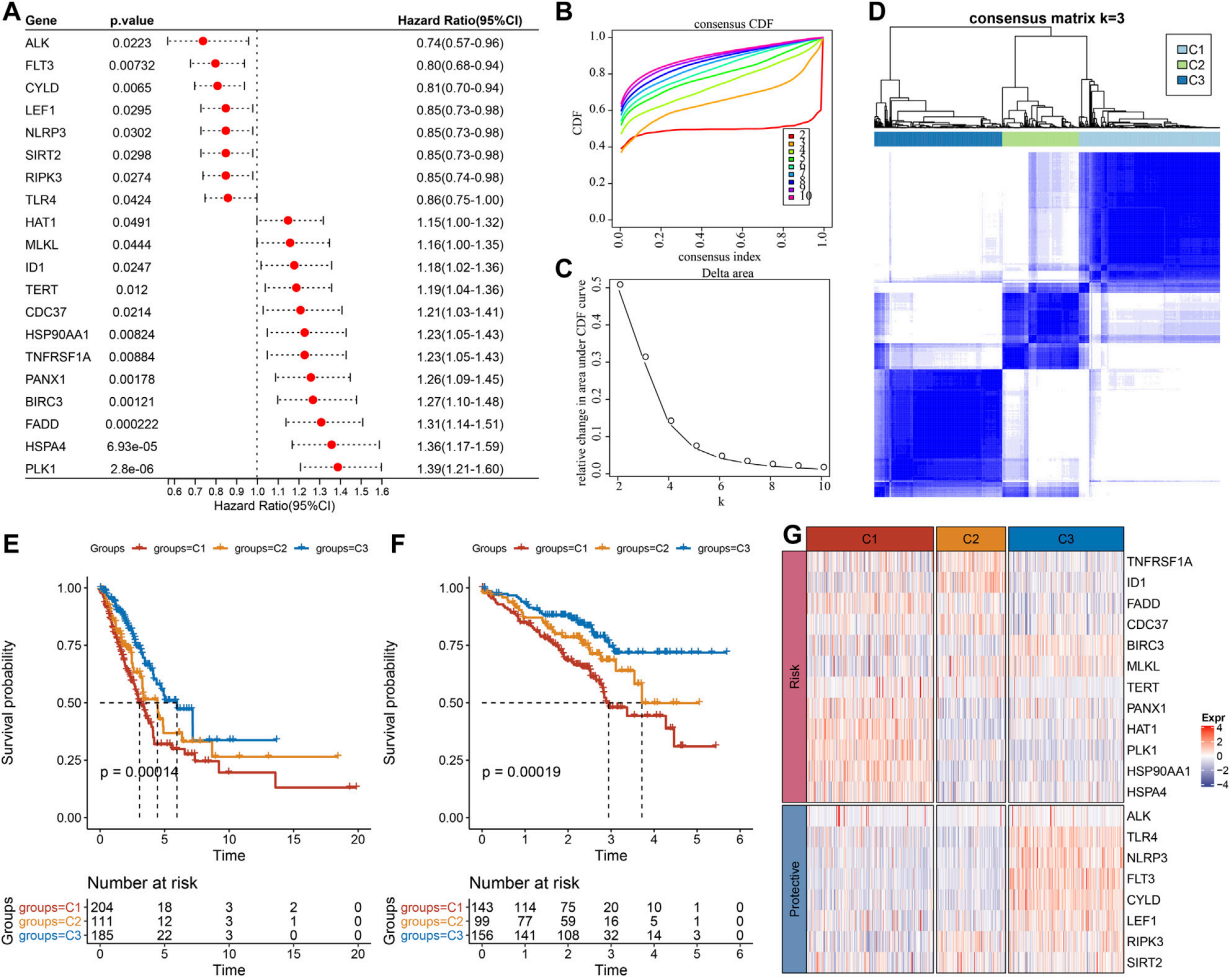
Molecular typing results according to the necroptosis-related genes. **(A)** Forest map of genes related to necroptosis with significant prognosis in TCGA cohort; **(B)** cumulative distribution function (CDF) curve of the samples in TCGA cohort; **(C)** The CDF Delta area curve of TCGA cohort sample highlights the relative change of the area under the CDF curve of each category number (k) in comparison with K-1. The horizontal axis is for k, and the vertical axis is for the relative change of the area under the CDF curve; **(D)** Sample clustering Heatmap when consensus k = 3 in TCGA queue; **(E)** Kaplan-Meier (KM) curve of overall survival (OS) prognosis of three subtypes in TCGA cohort; **(F)** Prognostic variations in the three molecular subtypes in the GSE72094 cohort; **(G)** The heat map of the expression of necroptosis genes with significant prognosis in different subtypes of TCGA.

Firstly, the expression of necroptosis-related genes was taken from the expression matrix of TCGA, and 20 necroptosis genes with significant prognosis related to LUAD were selected (Figure 1A, *p* < 0.05). Patients were classified by consensus clustering in accordance with the expression profiles of these 20 genes. We finally determined that the optimal number of clusters was 3 as it gave us comparatively stable clustering outcomes (Figures 1B,C), i.e., k = 3 to get three separate molecular subtypes (Figure 1D). Further analysis revealed that there were major prognostic variations in the prognostic features of the three molecular subtypes (Figure 1E). In general, C3 showed a good prognosis, and the C1 subtype had a poor prognosis. In addition, for the GSE72094 data set, after molecular typing with the same method, it was found that there were major variations in prognosis (Figure 1F), similar to the training set. At the same time, the expression differences of these 20 necroptosis genes that were substantially linked with the prognosis in separate molecular subtypes of TCGA were compared (Figure 1G).

### Clinicopathological characteristics among molecular subtypes

**FIGURE 2:**
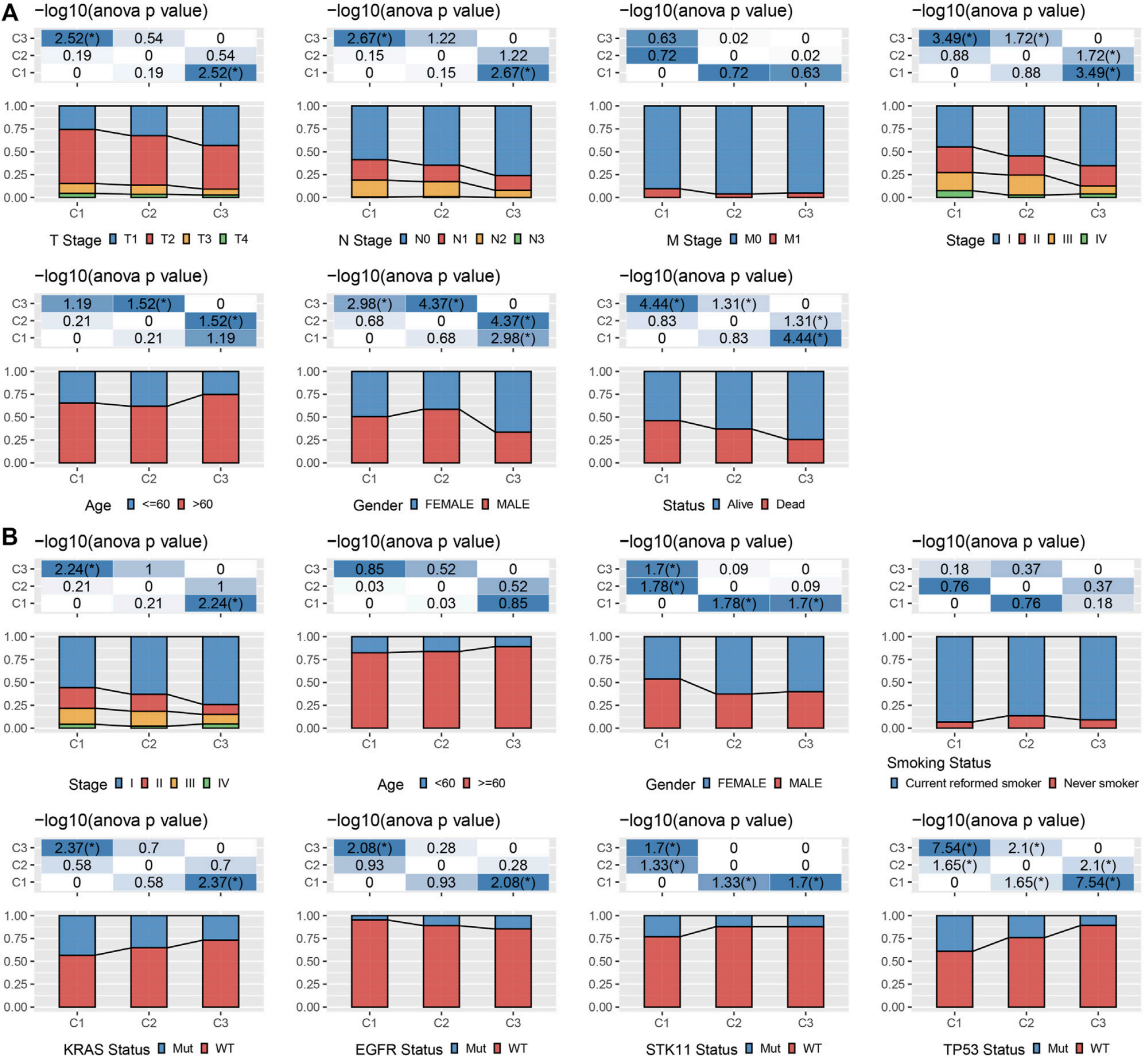
Clinicopathological properties of molecular subtypes. **(A)** The clinicopathological characteristics of molecular subtypes in TCGA cohort; **(B)** The clinicopathological characteristics of molecular subtypes of GSE72094 cohort; The lower and upper parts of the proportion are the statistical significance of the distribution difference in two pairs -log10 (*p*-value).

We kept on exploring the differences in clinicopathological characteristics in separate molecular subtypes present in the TCGA cohort. In the TCGA data set, there were variations in the distribution of diverse clinical features among the three molecular subtypes. It could be observed that the C1 subtype had a high clinical grade, and male patients accounted for a large proportion of C1 and C2 subtypes (Figure 2A). Moreover, we also compared the clinicopathological characteristics of various molecular subtypes in the GSE72094 cohort and observed that the proportion of Stage II in the C1 subtype was substantially greater than that of the other two subtypes, and the proportion of Stage II was considerably reduced in comparison with that of the other two subtypes. KRAS, STK11, and TP53 gene mutations in patients with the C3 subtype were considerably reduced in comparison with those in patients with C1 and C2 subtypes, and EGFR gene mutations were significantly more than those in patients with C1 and C2 subtypes (Figure 2B).

### Mutation characteristics among molecular subtypes

**FIGURE 3:**
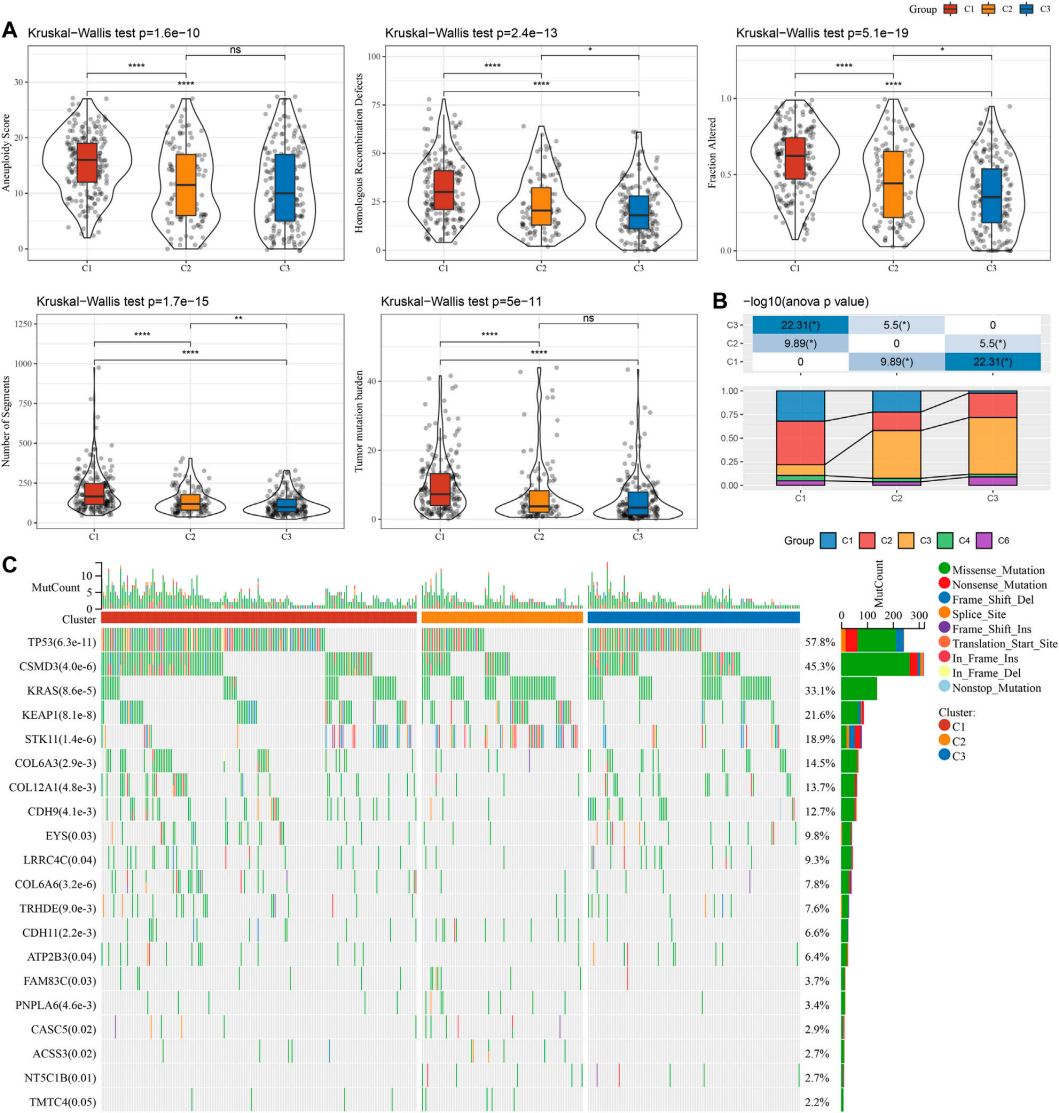
Genomic changes of molecular subtypes in TCGA cohort. **(A)** The differences in Homologous Recombination Defects, Aneuploidy Score, Fraction Altered, Number of Segments, and Tumor mutation burden among the molecular subtypes of TCGA cohort were compared; **(B)** Comparison of three molecular subtypes and immune molecular subtypes; **(C)** Somatic mutations in three molecular subtypes (chi-square test). *p < 0.05, **p < 0.01, ***p < 0.001, and ****p < 0.0001.

This report also explained the variations of genomic alterations in the three molecular subtypes in the TCGA cohort. Firstly, the molecular characteristic information of TCGA was obtained from the previous pan-cancer research (Thorsson et al., 2018). Among them, the C1 subtype showed a higher Homologous Recombination Defects, Aneuploidy Score, Number of Segments, Fraction Altered, and Tumor mutation burden (Figure 3A). In addition, according to 160 different immune signatures, LUAD was divided into five immune subtypes, of which the best prognosis was observed in the immune subtypes C3 and C4 and C6 had the poorest prognosis. It was discovered that, of the three types of molecular subtypes defined in this study, the C3 subtype described in the previous study accounted for more of the C3 subtypes described when the relationship between these five immune subtypes and the three types of molecular subtypes described by us was compared (Figure 3B). In addition, based on the correlation analysis between gene mutation and molecular subtype, we concluded that there was a major link between molecular subtype and gene mutation. TP53, CSMD3, and KRAS, and other genes had numerous somatic mutations in LUAD, and the TP53 gene had the highest mutation frequency in the C1 subtype (Figure 3C).

### Immune characteristics among molecular subtypes

**FIGURE 4:**
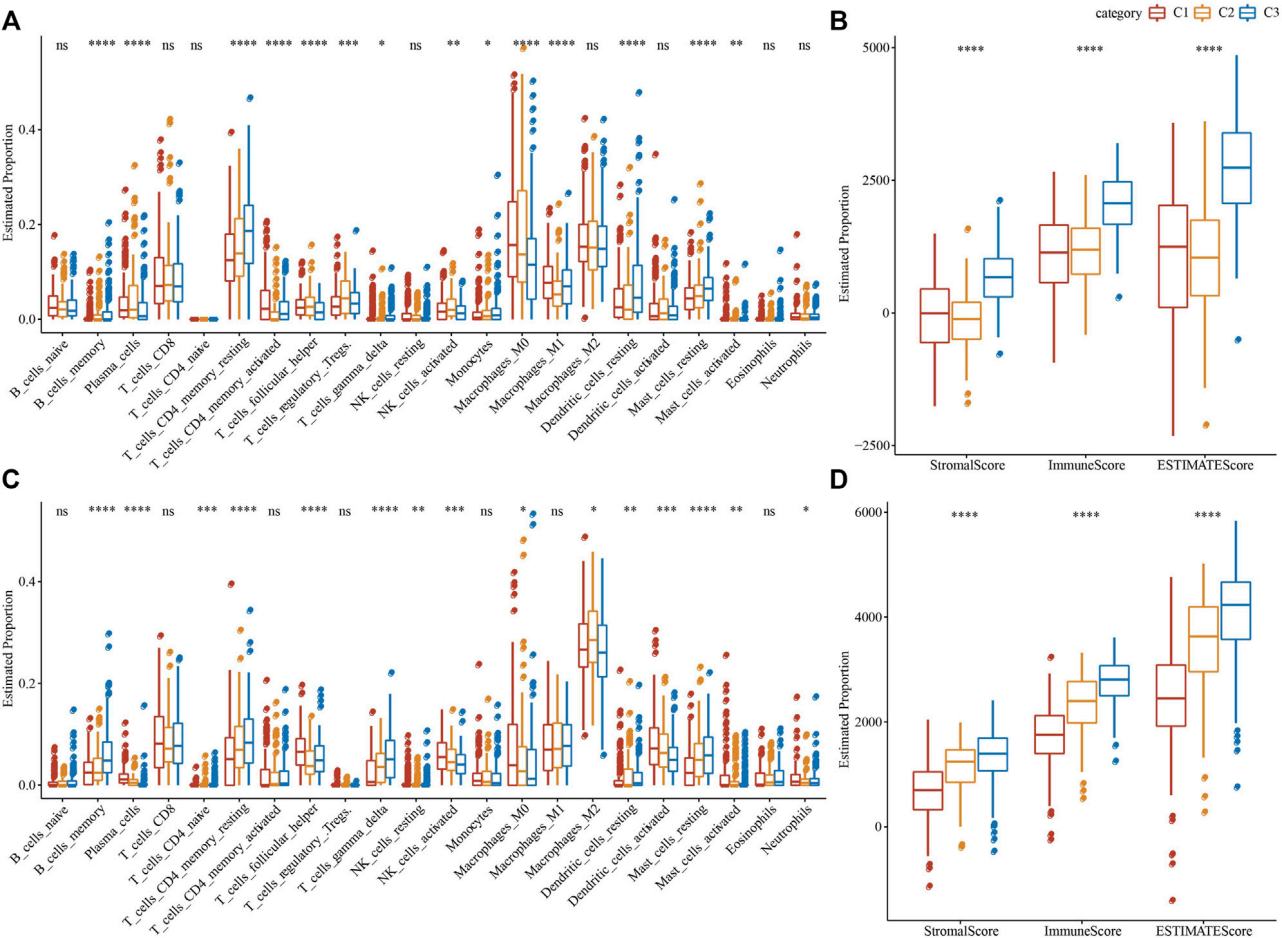
Proportion of immune cell components in two LUAD cohorts. **(A)** The variation of 22 immune cell scores among different molecular subtypes in the TCGA cohort; **(B)** The difference of ESTIMATE immune infiltration among different molecular subtypes in the TCGA cohort; **(C)** Differences in scores of 22 immune cells in various molecular subtypes in GSE72094 cohort; **(D)** Differences of ESTIMATE immune infiltration in various molecular subtypes in GSE72094 cohort. *p < 0.05, **p < 0.01, ***p < 0.001, and ****p < 0.0001.

To clearly understand the difference in immune microenvironment among subjects with various molecular subtypes, the level of immune cell infiltration in patients in the TCGA cohort was evaluated by the gene expression level in immune cells. Firstly, based on the relative abundance of 22 immune cells (Figure 4A), it was observed that most immune cell types had significant differences among subtypes. For example, macrophages of the M1 type were substantially more infiltrated in C1 and C3 subtypes than in C2, while regulatory T cells (Tregs) were substantially more infiltrated in C2 subtypes in comparison with the C1 and C3. Simultaneously, the “immune score” of the C3 subtype was increased in comparison with that of other subtypes, i.e., C1 and C2 subtypes, with higher immune cell infiltration (Figure 4B). In addition, by comparing the immune infiltration of the GSE72094 cohort (Figures 4C,D), a similar phenomenon to TCGA could be observed.

### Pathway analysis between molecular subtypes

**FIGURE 5:**
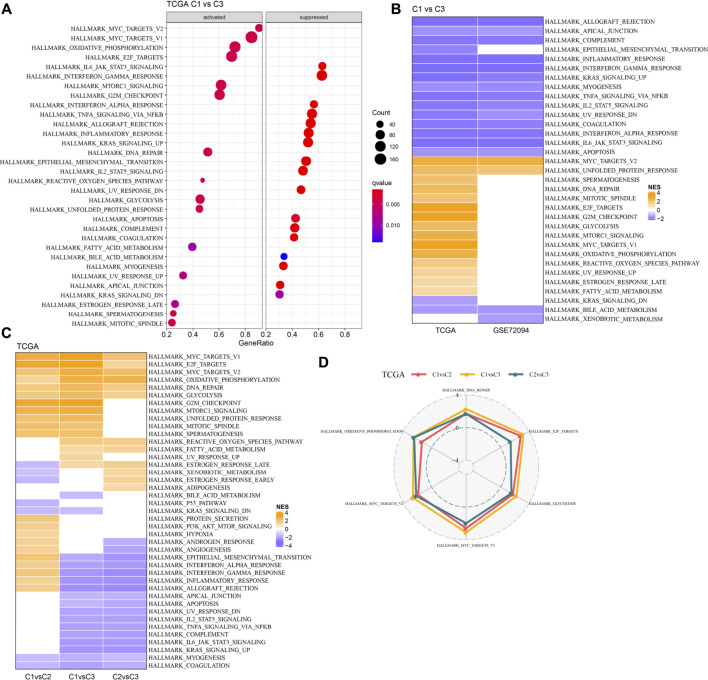
Pathway analysis between molecular subtypes. **(A)**. GSEA analysis results of C1 vs. C3 in TCGA cohort; **(B)** Bubble chart of GSEA analysis results of C1 vs. C3 subtypes in two LUAD cohorts; **(C)** Bubble chart of GSEA analysis results compared with different molecular subtypes in TCGA cohort; **(D)** Radar chart of C1 vs. C2 and C2 vs. C3 uniformly activated channels in TCGA queue.

GSEA analysis was done to identify the differentially activated pathways in various molecular subtypes. The outcomes revealed that in comparison with the C3 subtype, the C1 subtype was significantly enriched in 32 pathways in the TCGA cohort and 18 pathways in the GSE72094 cohort (Figures 5A,B). Simultaneously, through the comparative analysis of abnormal pathways in C1 and C3 subtypes in various LUAD cohorts, it was found that the activated pathways mainly included some cell cycle-related pathways, such as HALLMARK_UNFOLDED_PROTEIN_RESPONSE, HALLMARK_MYC_TARGETS_V2, HALLMARK_DNA_REPAIR, HALLMARK_MITOTIC_SPINDLE, etc., while the inhibited pathways mainly included some immune-related pathways, such as HALLMARK_INFLAMMATORY_RESPONSE, HALLMARK_INTERFERON_GAMMA_RESPONSE, HALLMARK_ALLOGRAFT_REJECTION, HALLMARK_COMPLEMENT, HALLMARK_INTERFERON_ALPHA_RESPONSE, etc. (Figure 5B). Through the comparative analysis of the pathways in C1 and C2, C1 and C3 subtypes, and the differences between C2 and C3 subtypes in the TCGA cohort (Figures 5C,D), it was found that the cell cycle-related pathways in C1 patients were activated on the whole, while the immune-related pathways were inhibited. Therefore, we inferred that the necroptosis genes used for molecular typing might play a critical role in the cell cycle-related pathways and the tumor microenvironment.

### Analysis of differentially expressed genes in molecular subtypes

**FIGURE 6:**
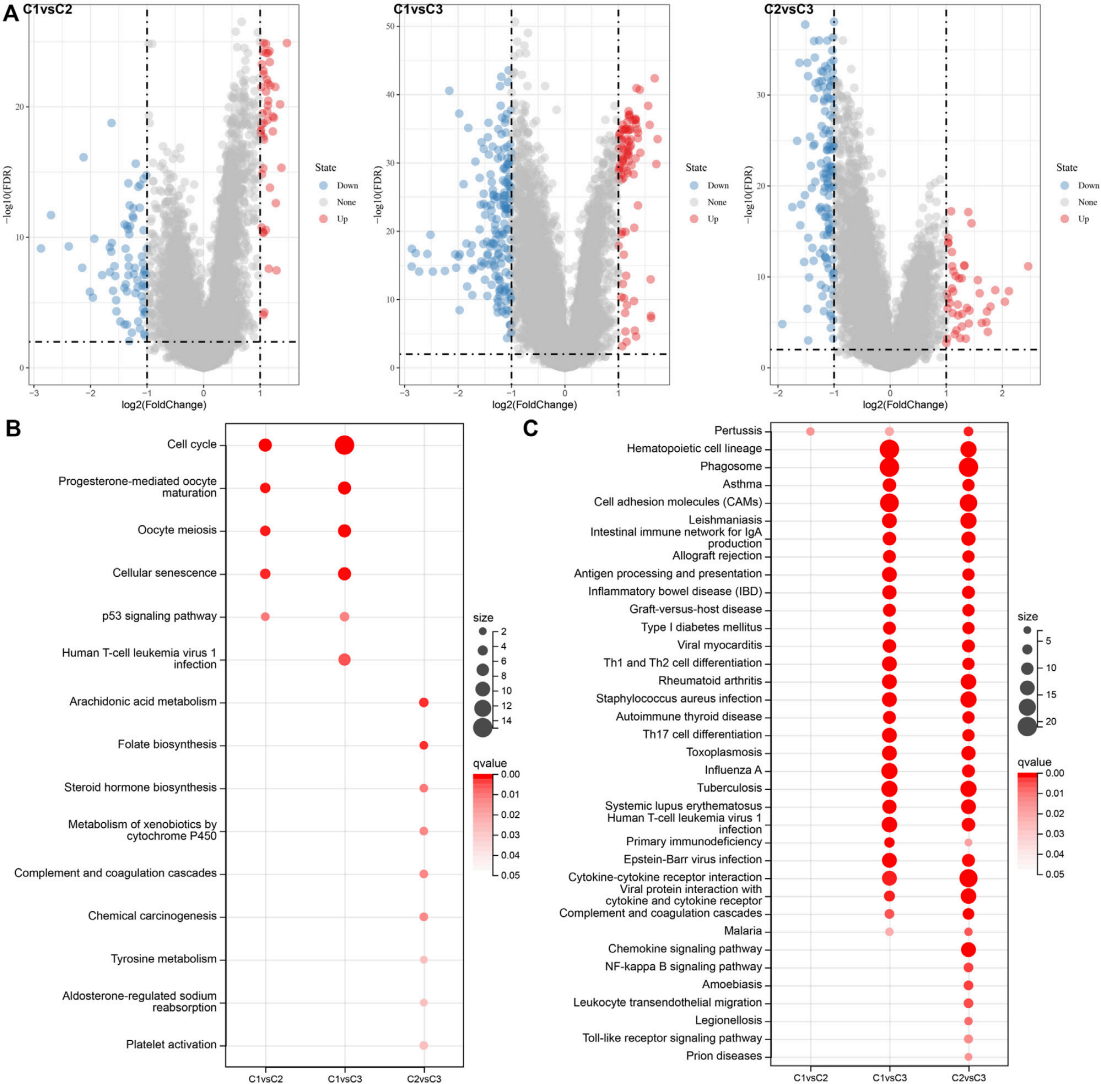
Differential expression analysis between molecular subtypes. **(A)** Volcano diagram of DEGs among TCGA molecular subtypes; **(B)** Bubble chart of KEGG function enrichment analysis results of differentially up-regulated genes among TCGA molecular subtypes; **(C)** Bubble chart of KEGG function enrichment analysis results of differentially downregulated genes among TCGA molecular subtypes.

In the analysis described above; three separate molecular subtypes were identified by the necroptosis genes with significant univariate prognosis. Next, the differentially expressed genes (DEGs) among C1 vs. C2, C1 vs. C3, and C2 vs. C3 subtypes were calculated by using the limma package. Firstly, there were 119 DEGs between the subtypes C1 and C2, including 46 highly expressed and 73 genes with low expression. Secondly, among the DEGs of C1 and C3 subtypes, there were 88 up-regulated genes and 183 down-regulated genes. Finally, among the DEGs between C2 and C3 subtypes, there were 45 up-regulated genes and 140 downregulated genes (Figure 6A). The Kyoto Encyclopedia of Genes and Genomes (KEGG) pathway enrichment analysis of differentially up-regulated genes among C1 vs. C2, C1 vs. C3, and C2 vs. C3 subtypes was performed by the R software package clusterprofiler. The results showed that C1 vs. C2 and C1 vs. C3 subtypes were substantially enriched in some pathways linked with cell cycle such as cellular senescence, cell cycle, p53 signaling pathway, etc. While the C2 vs. C3 subtype was significantly enriched in metabolic-related pathways (Figure 6B). Similarly, based on the KEGG pathway enrichment analysis of differentially downregulated genes among C1 vs. C2, C1 vs. C3, and C2 vs. C3 subtypes, the results showed that there were fewer differential pathways among C1 vs. C2 subtypes, while there were more differential pathways among C1 vs. C3 and C2 v sC3 subtypes, especially among C2 vs. C3 subtypes, and the down-regulated genes among these subtypes were substantially enriched in some immune and inflammatory-related differential pathways (Figure 6C).

### Identification of key necroptosis genes

**FIGURE 7:**
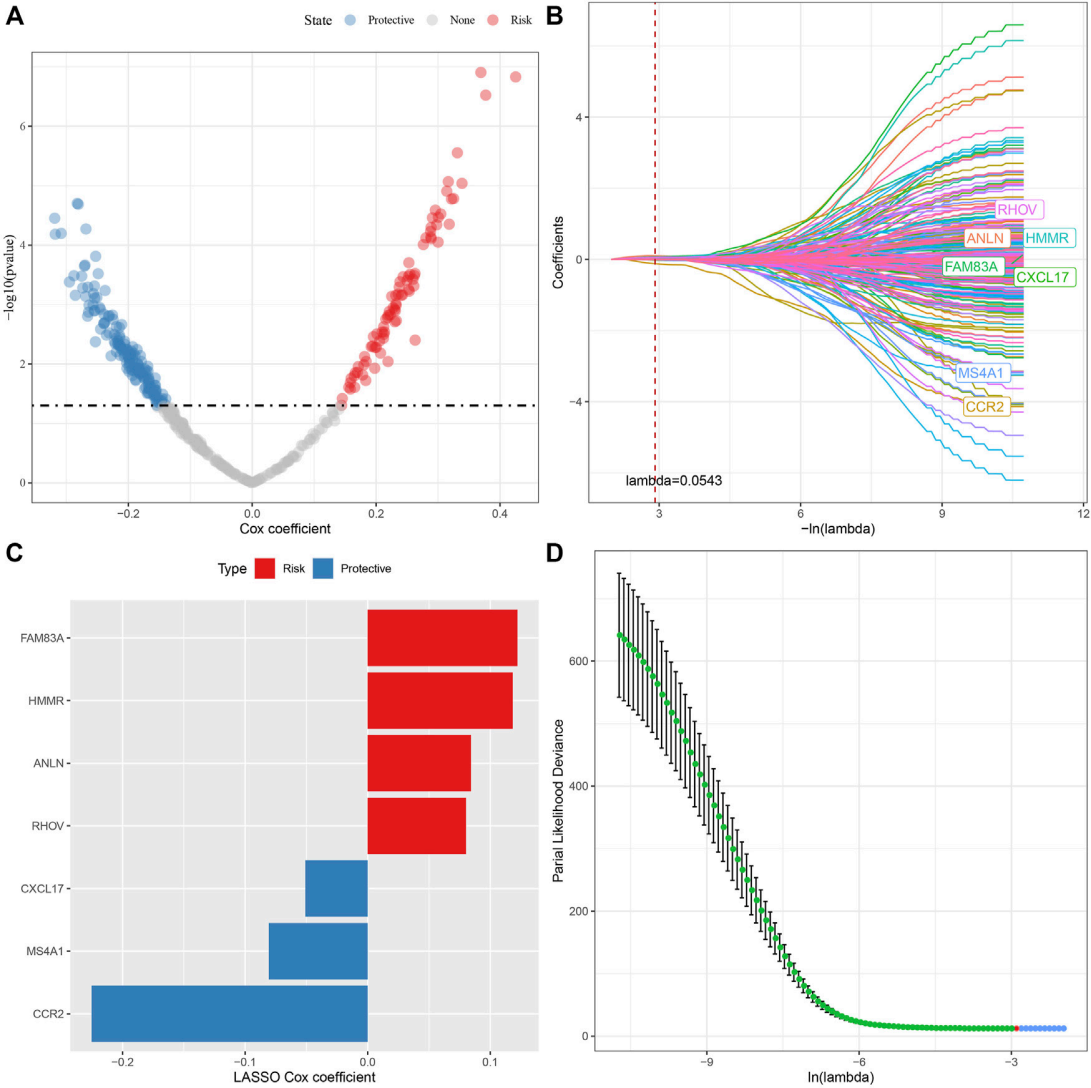
Lasso analysis of DEGs. **(A)** Analysis results of DEGs; **(B)** The locus of each independent variable changing with lambda; **(C)** Confidence interval under lambda; **(D)** Lasso coefficient distribution of the characteristics of genes linked with necroptosis.

405 genes were obtained by identifying DEGs among molecular subtypes. Next, these genes were assessed with univariate Cox regression analysis, along with the 242 genes that impacted the prognosis more (*p* < 0.05) were identified, including 84 “Risk” and 158 “Protective” genes (Figure 7A). Then, for Lasso regression, the “glmnet” R package was utilized to select the proportion of genes used to build risk models among the 242 genes with significant prognosis. Each independent variable’s change track was first examined. The number of independent variable coefficients that are progressively heading to 0 rose as the lambda value increased (Figure 7B). 10-fold cross-validation was utilized for creating the model, and the confidence interval under each lambda was assessed. The value of lambda = 0.0543 indicated the optimal output (Figure 7C). Therefore, we selected FAM83A, HMMR, ANLN, RHOV, CXCL17, MS4A1, and CCR2 as the related genes of necroptosis phenotype that affected the prognosis when lambda = 0.0543 (Figure 7D).

### Establishment and verification of risk model

**FIGURE 8:**
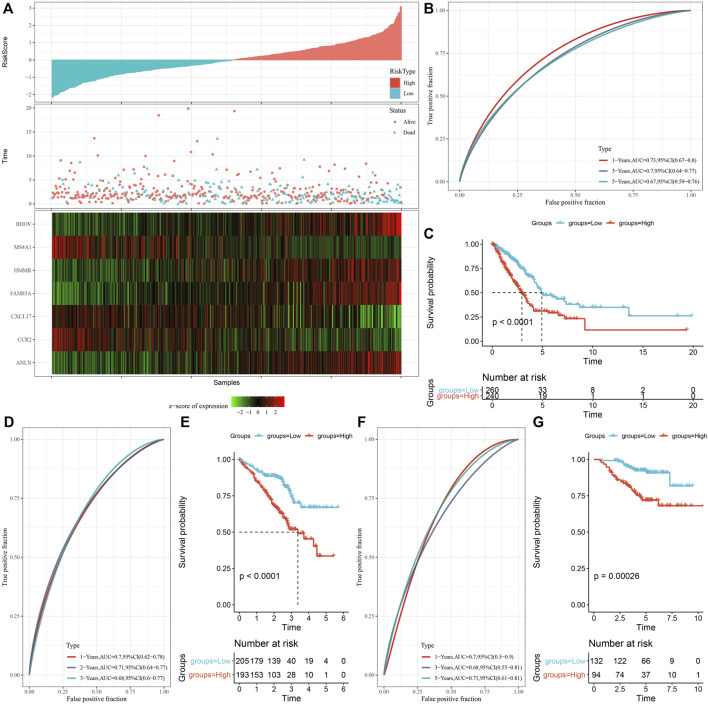
Establishment and verification of risk model. **(A)** RS, survival time, survival status, and expression of necroptosis-related genes in TCGA data set; **(B)** ROC curve and AUC classified by RS in TCGA data set; **(C)** KM survival curve distribution of RS in TCGA data set; **(D,E)** ROC curve and KM survival curve of RS in GSE72094 queue; **(F,G)** ROC curve and KM survival curve of RS in GSE31210 queue.

The prognostic RS related to apoptosis was calculated and normalized for each sample. At the same time, samples with RS greater than 0 were put in the RS-high group and samples having RS less than or equal to 0 were put in the RS-low group. A major difference was observed in the RS-high and -low groups (*p* < 0.001). Finally, 260 samples were put in the RS-high group, and 240 samples into the RS-low group. The RS distribution of patients in the TCGA cohort of the training set suggested that RS-high samples had a poor prognosis (Figure 8A). The ‘timeROC’ R package was utilized for assessing the Receiver Operating Characteristic (ROC) of RS prognosis classification, and the classification efficiency of prognosis prediction for 1, 3, and 5 years were analyzed respectively (Figure 8B). The outcomes revealed a high area under curve (AUC) values of prognosis prediction of the model for 1, 3, and 5 years, which were 0.73, 0.7, and 0.67, respectively. Finally, patients with higher RS showed worse overall survival in the training cohort (Figure 8C). For confirming the robustness of the clinical prognosis model prediction of necroptosis-related genes, it was verified in GSE72094 and GSE31210 cohorts. The RS of patients was calculated following the same method and samples were divided into high group when RS > 0 and samples were put in low group when RS < 0. The validation cohort had outcomes similarly to those of the training set. The prognosis of high RS was poor, while that of low RS was good (Figures 8D–G).

### Riskscore in different clinicopathological characteristics

**FIGURE 9:**
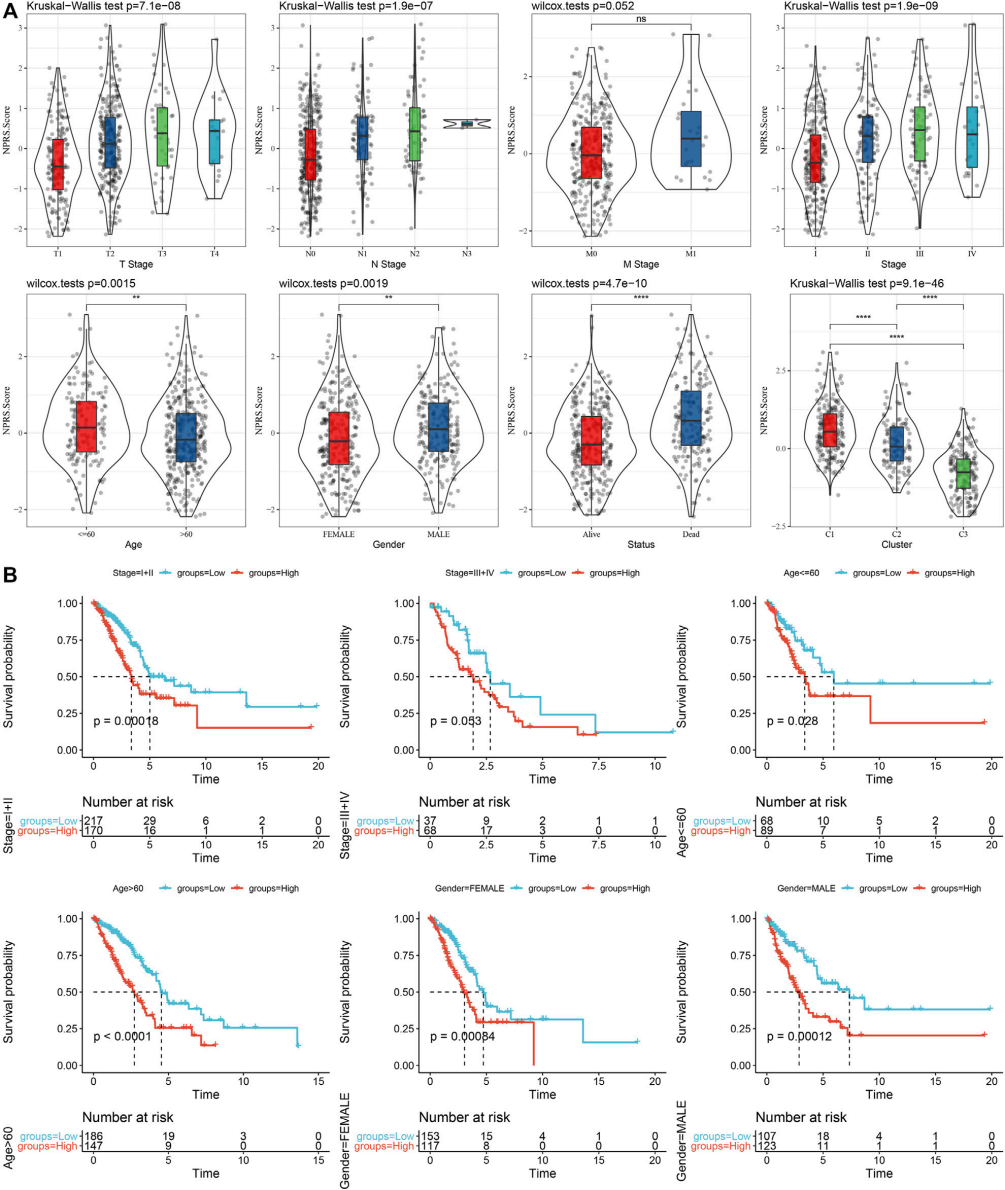
RS in different clinicopathological characteristics. **(A)** The difference of RS between different clinicopathological groups of the TCGA cohort; **(B)** KM curve between RS-high and -low groups among different clinicopathological groups of the TCGA cohort.

We discovered that the RS of patients with late T Stage, N Stage, M Stage, and Stage was considerably greater in comparison with that of patients with early stage by the comparison of RS distribution among the groups of clinicopathological features in the TCGA cohort. Additionally, we discovered that male patients had a higher RS. Between molecular subtypes, RS was compared and examined. When compared to the RS of C3 molecular subtype with a favorable prognosis, the RS of the C1 subtype with a much worse prognosis was significantly higher (Figure 9A). In addition, based on the comparative analysis of the prognosis differences between different clinicopathological characteristics groups in the TCGA cohort in the RS-high and -low groups defined by us, our risk groups also had good results in different clinical groups, proving the reliability of our risk groups (Figure 9B).

### Characteristics of immune/pathways between riskscore groups

**FIGURE 10:**
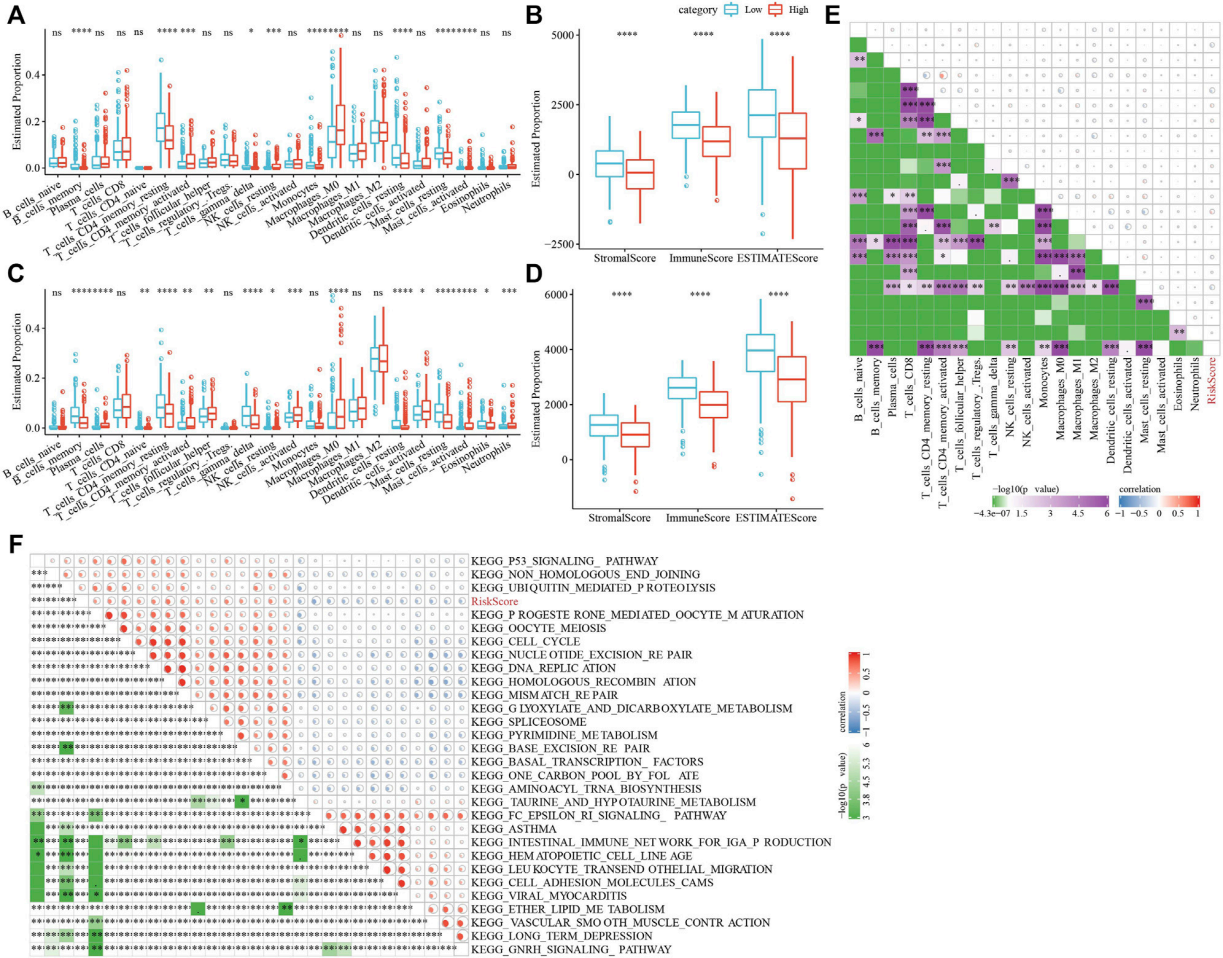
Characteristics of immune/pathways among the RS groups. **(A)** Proportion of immune cells components in TCGA cohort; **(B)** Proportion of immune cells components calculated by ESTIMATE software in TCGA cohort; **(C)** Proportion of immune cells components in TCGA cohort; **(D)** Proportion of immune cells components calculated by ESTIMATE software in TCGA cohort; **(E)** Correlation analysis between 22 immune cell components and RS in TCGA cohort; **(F)** The results of correlation analysis between KEGG pathway and RS whose correlation with RS is greater than 0.45. *p < 0.05, **p < 0.01, ***p < 0.001, and ****p < 0.0001.

To clarify the variation in the immune microenvironment of patients in the RS group, the relative abundance of 22 immune cells in RS-high and -low groups in the TCGA cohort was compared. There were significant variations in 10 immune cells present in the RS-high and -low groups (Figure 10A). Such as, the abundance of resting CD4 memory T cells in the RS-low group were much higher in comparison with that in the RS-high group, while the abundance of activated CD4 memory T cells in the RS-low group was considerably lower when compared with the RS-high group. At the same time, the ESTIMATE was used for evaluating the immune cell infiltration. It was found that “the estimated immune sub-group” had higher immune infiltration (Figure 10B). This phenomenon was also observed in the GSE72094 cohort (Figures 10C,D).

Then, we studied the link of RS with 22 immune cell components in the TCGA queue and observed that RS and resting CD4 memory T cells, activated CD4 memory T cells, and resting dendritic cells along with nine others (Figure 10E).

To analyze the link of RS with the biological role of distinct samples, we chose the gene expression profile relating to the LUAD samples in the TCGA cohort and used the GSVA R package for single sample Gene Set Enrichment Analysis (ssGSEA). The score of individual samples on various functions was measured to get the ssGSEA score of individual functions related to each sample. After studying the link between these functions and calculating the RS, functional pathways greater than 0.45 were selected, from which we could see that RS and KEGG_CELL_Cycle and other cell cycle-related pathways showed a positive correlation (Figure 10F).

### Differences in immunotherapy/chemotherapy between riskscore groups

**FIGURE 11:**
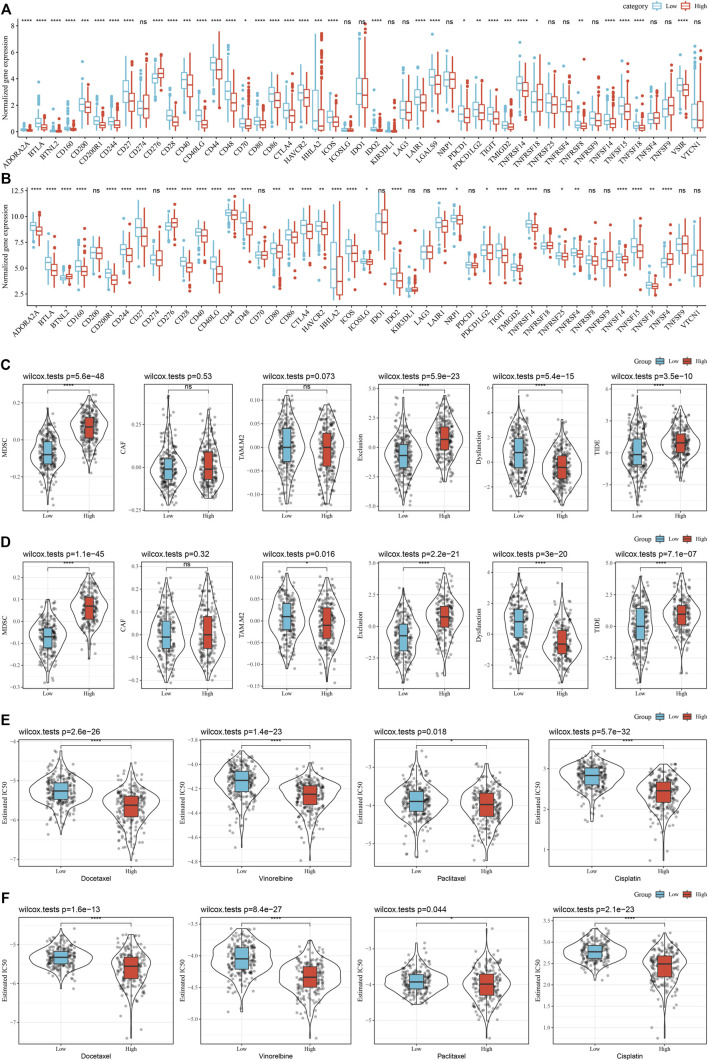
(Continued).

In addition, whether there were differences in immunotherapy between RS groups in the TCGA cohort were analyzed. First, we did a comparison of the expression of immune checkpoints among RS groups and found that most immune checkpoint genes were differentially expressed in RS groups. On the whole, the differential expression of immune checkpoint genes, such as CTLA4, PDCD1, in the RS-low group was considerably increased in comparison with that in the RS-high group (Figure 11A). In addition, by evaluating the possible clinical impact of immunotherapy in the RS-high and -low groups, we observed that in the TCGA cohort, the RS-high group had an increased TIDE score, suggesting that the possibility of immune escape for the RS-high group was more and that of benefiting from immunotherapy was less (Figure 11C). The scores of MDSC and T cell rejection were increased in the RS-high group, which might be a factor leading to the low benefit of immunotherapy in the RS-high group. Furthermore, the response of the RS group in the TCGA cohort to traditional chemotherapy drugs, such as docetaxel, vinorelbine, paclitaxel, and cisplatin was also analyzed. We discovered that the RS-high group showed more sensitivity to the stated drugs than the RS-low group (Figure 11E).

At the same time, the differences in immunotherapy and chemotherapy among RS groups in the GSE72094 cohort were analyzed, and the same phenomenon as that in the TCGA cohort was observed (Figures 11B,D,F).

### Riskscore combined with clinicopathological characteristics for improving the prognosis model and survival prediction

**FIGURE 12:**
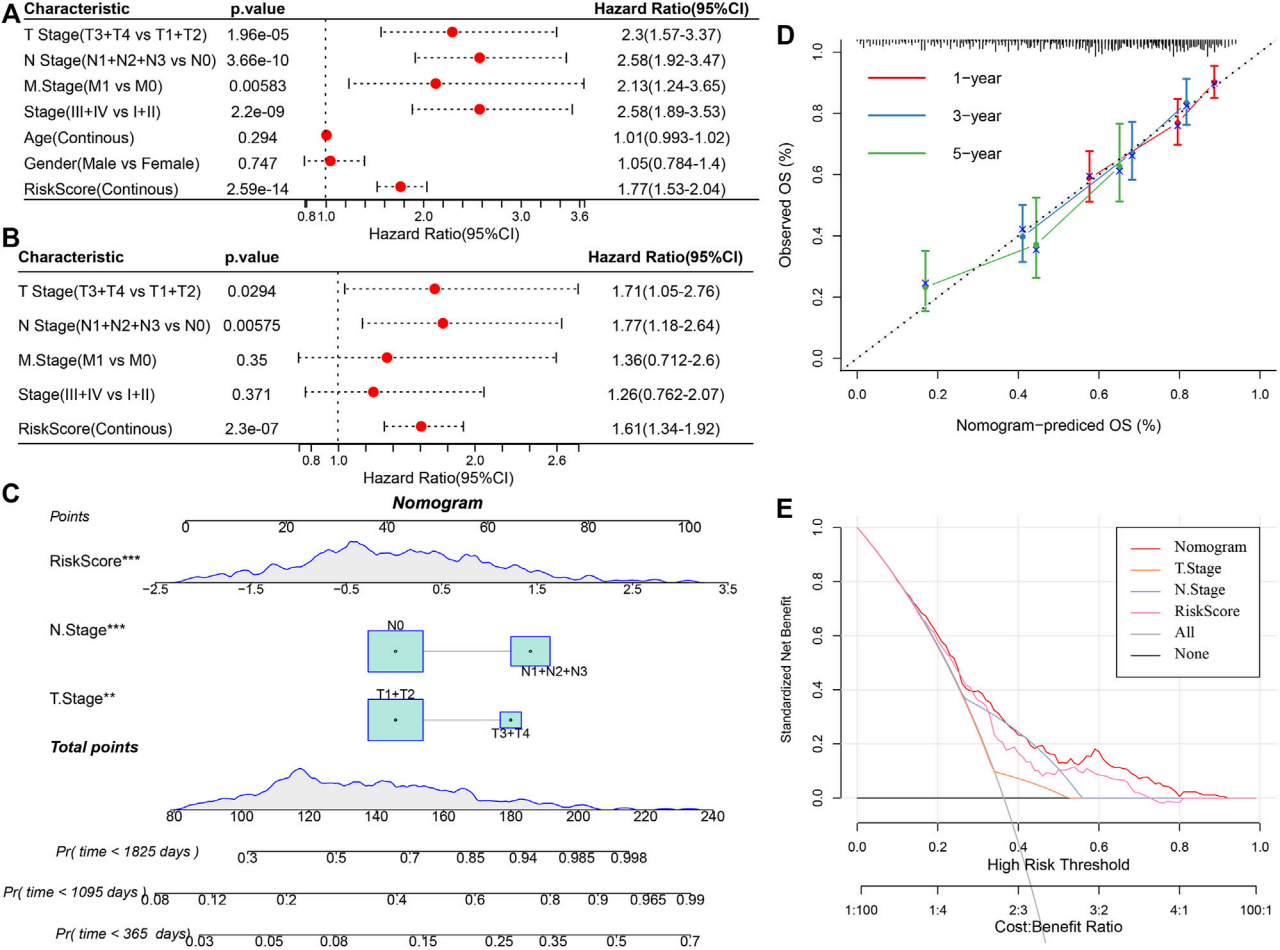
Improvement of prognosis model and survival prediction. **(A,B)** univariate and multivariate Cox analysis of RS and clinicopathological characteristics; **(C)** Nomogram model; **(D)** Calibration curve of nomogram in 1, 3, and 5 years; **(E)** Decision curve of the nomogram.

Univariate and multivariate Cox regression analysis of RS and clinicopathological features showed that RS was the most significant prognostic factor (Figures 12A,B). For risk assessment quantification and survival probability of patients with LUAD, a nomogram was established (Figure 12C) in combination with RS, N Stage, T Stage, and other clinicopathological characteristics. The model results showed the greatest effect of RS on survival rate prediction. We evaluated the model for its prediction accuracy using a calibration curve, it could be observed that the predicted calibration curve of the three calibration points in 1, 3, and 5 years was near the standard curve (Figure 12D), showing the nomogram’s good prediction ability. Moreover, the decision curve analysis (DCA) was also utilized for evaluating the model’s reliability. It could be seen that the benefits of RS and nomogram were considerably increased when compared with that of the extreme curve. Compared with other clinicopathological characteristics, the nomogram showed the strongest ability to predict survival, followed by RS (Figure 12E).

## Discussion

Necroptosis is a type of cell death related to the morphological characteristics of necrotic cells and its intrinsic signal transduction is like that of apoptotic cells. Nevertheless, necroptosis and apoptosis are different mechanisms that help in the inhibition of tumor development and metastasis (Fu et al., 2013; Lawlor et al., 2015; Newton, 2015). Numerous research conducted since the word “necroptosis” was first proposed have revealed that necroptosis can prevent tumor growth and metastasis, suggesting that it can be used for the treatment of cancer (Li et al., 2020a; Park et al., 2020; Tan et al., 2020). However, molecular typing of LUAD according to genes linked with necroptosis has not been reported. Based on necroptosis, cluster analysis was done using LUAD samples provided by the TCGA and GEO data sets, and we obtained three molecular subtypes C1, C2, and C3 of LUAD. C1 had a worse prognosis than C3, whereas C3 had a better prognosis. The matrix and immune cells enlisted and activated in the microenvironment associated with the tumor determine the tumor cells in LUAD. Immune cells and immune-related molecules also infiltrate the tumor microenvironment, which is where tumor cells proliferate, develop, and prepare for metastasis (Seong et al., 2020; Sprooten et al., 2020; Ma et al., 2021). Therefore, the variations in the immune microenvironment in subjects with different molecular subtypes were also observed, which showed that the immune score of the C3 subtype was increased in comparison with that of other subtypes, indicating that the C3 subtype had relatively high immune cell infiltration. This was supported by our prior study showing that C3 had a good prognosis and the overall survival rate of patients with a high immune score was more in comparison with that of patients having a low immune score. This finding indicates that from the beginning of tumor formation, LUAD patients with higher immune scores may have stronger adaptive immune responses than those with lower immune scores (Ma et al., 2021). Therefore, the higher immune cell content and an immune score of C3 may be one of the guarantees of a good prognosis.

Then, we calculated RS and constructed a risk model, in which RS-high samples had a worse prognosis. In addition, the evaluation of potential clinical effects of immunotherapy in RS-high and -low groups showed that the RS-high group had a higher score and a higher possibility of immune escape. In other words, in comparison with the RS-low group, the patients of the RS-high group were observed to have a worse prognosis and up-regulated expression of immune checkpoints. They were more suitable for immunotherapy and were more likely to benefit from it.

Based on the role of necroptosis in the regulation of tumor immunity, we carried out the ssGSEA to find the immune status of various RS groups. Immune cells (resting CD4 memory T cells, memory B cells, and resting dendritic cells) were mostly active in the RS-low group, among these, some were closely linked with necroptosis. But, CD8 T cells had no significance between high- and low-group. Necrotic cells could provide dendritic cells with tumor-specific antigens and inflammatory cytokines for antigen cross initiation (Sprooten et al., 2020). These outcomes indicate the possible involvement of necroptosis in the progression of LUAD by tumor immunity regulation.

In addition, the function of abnormal pathways in the C1 and C3 subtypes was analyzed, and the results showed that the activated pathways mainly included some cell cycle-related pathways, such as HALLMARK_MYC_TARGETS_V2. The relationship between cell cycles and necroptosis is inseparable. MYC pathway is one of the most significant signal pathways in the process of necroptosis. In addition, the MYC transcription factor has been shown in other studies to inhibit the formation of anti-necrotic protein of the RIPK1-RIPK3 complex (Seong et al., 2020), which fully demonstrates the reliability of our typing results. Different subtypes do have great differences in the process of necroptosis.

Studies have shown that FAM83A and FAM83A-AS1 are upregulated in LUAD in comparison with the adjacent healthy tissues. This high expression indicates poor survival and more advanced clinical stages (Wang et al., 2021a). Moreover, several studies have shown that FAM83A can be used as a prognostic characteristic and potential oncogene of LUAD (Zhang et al., 2019; Gan et al., 2020; Yu et al., 2020; Song et al., 2021). In this study, seven genes were identified as prognostic genes related to the phenotype of necroptosis, and FAM83A was one of them. In addition, these six genes (HMMR (Li et al., 2020b; Li et al., 2021), ANLN (Zhang et al., 2020; Deng et al., 2021), RHOV (Wang et al., 2021b; Zhang et al., 2021), CXCL17 (Liu et al., 2020; Wang et al., 2022), MS4A1 (Ma et al., 2020; Song et al., 2020), and CCR2 (Liu and Wu, 2021; Wan et al., 2021)) have also been studied to support their use as potential prognostic biomarkers and possible immunotherapeutic targets related to LUAD, but our research supports their involvement in the incidence and development of LUAD from the perspective of cell necroptosis.

The classification based on the correlation between necroptosis provides a novel insight for research on LUAD. In addition, nomograms were established in the LUAD cohort based on RS and clinicopathological characteristics. Compared with other clinicopathological characteristics, RS in this nomogram had significant advantages in accurately predicting the survival rate of LUAD and greatly enhanced the clinical application of gene risk typing linked with necroptosis. Therefore, the typing proposed in this study is new and meaningful, and it is found that necroptosis-related genes may be involved in it.

Nevertheless, this study has certain deficiencies and limitations. First, for external validation, the addition of more clinical databases is preferable. Moreover, further experimental evidence is still needed to confirm the conclusions of this paper. For example, experiments are needed to verify the expression differences of genes linked with necroptosis in three separate molecular subtypes. Finally, experiments should verify that the necroptosis-related genes in different subtypes have an impact on tumor progression and prognosis, and the specific study of their possible interaction and regulation mechanism needs to be further studied. To overcome the shortcomings of this research, we will recollect and expand clinical samples in the follow-up work, perform more external experiments for verifying the efficacy of this model, and conduct large-scale independent studies in the future to confirm the efficacy of this risk classification.

In summary, the predictive attributes of genes linked with necroptosis have the ability of independent prognostic prediction of LUAD patients, assist in elucidating the mechanism and process of necroptosis genes in LUAD, and provide LUAD patients with immunotherapy guidance, but additional experimental confirmation is still required in the future.

## Conclusion

In a word, the stable molecular subtypes were identified by using the related genes of necroptosis through consensus clustering. Then, we chose a total of seven genes linked with the prognosis of necroptosis by analyzing the DEGs among the molecular subtypes and Lasso. Additionally, the RS model was created based on the prognosis-related genes of necroptosis. The model had strong robustness, which was independent of clinical-pathological characteristics, and played a stable predictive effect in independent data sets. Finally, we combined RS with clinicopathological characteristics to further improve the prognosis model and survival prediction. The model had high prediction accuracy and survival prediction ability.

## Data Availability

The datasets presented in this study can be found in online repositories. The names of the repository/repositories and accession number(s) can be found in the article/supplementary material.
